# Evaluation of Modeled Hyperspectral Infrared Spectra Against All‐Sky AIRS Observations Using Different Cloud Overlap Schemes

**DOI:** 10.1029/2022EA002245

**Published:** 2022-07-01

**Authors:** Tianhao Le, Vijay Natraj, Amy J. Braverman, Yuk L. Yung

**Affiliations:** ^1^ Division of Geological and Planetary Sciences California Institute of Technology Pasadena CA USA; ^2^ Jet Propulsion Laboratory California Institute of Technology Pasadena CA USA

**Keywords:** radiative transfer, cloud overlap, model‐AIRS intercomparison, first Wasserstein distance

## Abstract

Hyperspectral infrared sounding contains information about clouds, which plays an important role in modulating Earth's climate. However, there is a great deal of uncertainty in modeling the radiative effect of clouds due to its complex dependence on various parameters. Therefore, cloudy scenarios are often neglected in retrievals of infrared spectral measurements and in data assimilation. One‐dimensional radiative transfer (RT) models have a limited capability to represent the cloud three‐dimensional multilayer structure. This issue is typically resolved by using a multiple independent column approach, which is computationally demanding. Therefore, it is necessary to find a balance between computational speed and accuracy for infrared RT all‐sky radiance simulations. In this study, we utilize the Community Radiative Transfer Model with four different cloud overlap schemes and compare against observations made by the Atmospheric Infrared Sounder (AIRS) using a statistical metric called the first Wasserstein distance. Our results show that the average cloud overlap scheme performs the best and successfully predicts the overall probability distribution of brightness temperature over nonfrozen oceans for a wide range of wavelengths. The mean absolute differences are less than 0.7 K for 846 selected AIRS channels between 790 cm^−1^ and 1231 cm^−1^.

## Key Points

- Hyperspectral simulations, for more than 80,000 scenarios, using different cloud overlap schemes are compared with Atmospheric Infrared Sounder (AIRS) observations
- Average overlap scheme provides best results compared with AIRS observations
- First Wasserstein distance is a more suitable metric than the commonly used Pearson correlation for comparing probability distributions

## 1 Introduction

Clouds and their feedbacks play a fundamental role in climate changes (see, e.g., Bony et al., 2016; Hartmann & Larson, 2002; Lindzen et al., 2001; Su et al., 2017). Advancements in hyperspectral infrared sounding have provided us with a rich data set of daily global radiance measurements since the turn of the century with information content to sense cloud properties. The Atmospheric Infrared Sounder (AIRS) is a hyperspectral infrared sounder on board the NASA Aqua satellite in a sun‐synchronous orbit (Aumann et al., 2003) that makes more than 2 million observations each day. The atmospheric state (profiles of temperature, humidity, trace gas concentration, and cloud parameters) can be retrieved from the radiance spectrum. The radiance spectrum can also be directly used in weather forecasting for data assimilation (Collard & McNally, 2009).

However, data from hyperspectral infrared sounders appear to be underutilized for the study of clouds. One of the reasons for this is that modeling cloud radiative transfer (RT) effects is complicated since clouds reflect solar radiation and emit longwave radiation. Further, the radiative effect of clouds depends on the cloud altitude, type, particle size, and overlap details (Faijan et al., 2012; McNally, 2009; Pavelin et al., 2008; Prates et al., 2014; Yi et al., 2016). Another reason is that for use in data assimilation, the RT model requires a combination of sufficient accuracy, efficiency, and Jacobian, tangent‐linear and adjoint model capabilities.

Uncertainties in RT modeling of cloudy atmospheres arise not only from lack of adequate knowledge of cloud optical properties but also because the vertical distribution of liquid and ice clouds is not known. The vertical cloud distribution is modeled by making assumptions about cloud overlap. Typical cloud overlap assumptions include maximum, random, and maximum‐random overlap. Geer et al. (2009) developed an average overlap scheme for microwave RT. In all cases, radiances are computed for several atmospheric “columns” with the effective radiance calculated using a weighted average of the individual column radiances. The columns are constructed from the horizontal cloud fraction at each atmospheric level (which is assumed to be known). Different overlap assumptions then determine how the cloudy layers are stacked in the vertical. In the case of maximum overlap, all the cloudy layers are concentrated in the same columns as much as possible. For random overlap, the cloudy layers are distributed randomly across the columns. Maximum‐random overlap assumes that the cloudy columns are maximally overlapped in adjacent vertical layers that are both cloudy but randomly distributed where there is a cloud‐free layer in between. Average overlap assumes an average cloud fraction over the whole vertical profile with weight based on the total hydrometeor densities (e.g., liquid/ice cloud, rain, and snow). Hogan and Illingworth (2000) introduced an exponential‐random overlap scheme, where the total cloud cover is given by a weighted average of the maximum‐random and random overlap estimates of cloud cover. The weight is a function of the grid resolution in the general circulation model.

The drawback of the maximum and random overlap methods is that they are based on geometric assumptions that are too simplified to handle multilayered clouds (Tian & Curry, 1989). Maximum‐random overlap, on the other hand, is more realistic; however, about 10–100 columns are required to accurately represent typical cloudy atmospheric scenarios (Chen et al., 2013). Therefore, using the maximum‐random overlap assumption significantly increases the runtime of RT models, necessitating the use of fast and accurate cloud overlap methods. The hydrometeor‐weighted average overlap approach has been shown to reduce errors by 40% in areas of clouds and precipitation (Geer et al., 2009) with the usage of just two columns. O’Dell et al. (2007) presented an alternative two and three independent column scheme for microwave RT, which they referred to as the “optimal” approach. The two‐column optimal scheme is more accurate than approaches with equally weighted columns but incurs ∼75% more computational cost because it requires an extra RT calculation.

Recent studies have made great progress in the usage of all‐sky microwave and infrared radiances for assimilation (Geer et al., 2018, 2019). Geer et al. (2019) tested a multiple independent column approach under the maximum‐random overlap assumption in the RT for TOVS (RTTOV) model. However, this approach is computationally demanding, taking about 34 times longer than clear sky RT in the European Center for Medium‐Range Weather Forecasting (ECMWF) system (Geer et al., 2019). Therefore, we need to find a balance between computation burden and accuracy for all‐sky infrared radiance assimilation. In this study, we evaluate the Community Radiative Transfer Model (CRTM) using four different cloud overlap schemes.

In order to evaluate the performance of CRTM for all‐sky radiance simulations, we simulate tens of thousands of scenarios and compare against AIRS observations by looking at the probability distribution function of the difference between the surface temperature (ST) (obtained from European Centre for Medium‐Range Weather Forecasting (ECMWF) model estimates) and the brightness temperature in several channels. This difference is a measure of the radiometric effect of clouds. Aumann et al. (2018) used Pearson correlation of brightness temperature histograms to evaluate RT model performance. This work differs from the Aumann et al. (2018) work in four important ways. First, the data used here have a much better spatiotemporal match with AIRS observations (see Section 2.2). Second, we perform simulations for about 30 times more scenarios. Third, we perform model‐observation comparisons for 846 selected AIRS channels between 790 cm^−1^ and 1,231 cm^−1^ compared to the two channels used by Aumann et al. (2018). Fourth, we demonstrate that the Pearson correlation is not an ideal approach to compare probability distributions (see Section 2.4). Instead, we utilize a statistical metric, called the first Wasserstein distance, to quantitatively measure how far model probability distributions deviate from AIRS observations. Compared to the Pearson correlation technique, the first Wasserstein distance is less sensitive to the choice of histogram bins and provides a better characterization of the overall shape of the distribution.

The paper is organized as follows. In Section 2, we describe the data set and relevant models used in this work. We also introduce the first Wasserstein distance statistical metric that is used for the model‐measurement intercomparison. Section 3 summarizes the simulation scenarios used in this study and discusses the evaluation of all‐sky CRTM simulated radiances under different cloud overlap schemes against AIRS observations. The major findings are described in Section 4.

## 2 Relevant Data Sets and Methods

### 2.1 AIRS

AIRS is a grating array spectrometer covering the thermal infrared and shortwave infrared spectral range with 2,378 channels. The instrument spectral resolving power is $\frac{\nu }{\delta \nu }$  = 1,200. The noise is typically smaller than 0.2 K. The nadir footprint of AIRS is 13.5 km from a 705 km orbit with scans of about ±49.5° from nadir (Aumann et al., 2003). AIRS observations in the infrared region enable atmospheric temperature and water vapor vertical profile retrieval.

We used AIRS observations between 31 October 2018 21:00 UTC and 01 November 2018 09:00 UTC. Land and frozen ocean scenarios are excluded to avoid the possibility of introducing errors due to biases in modeled surface albedos. Overall, 82,271 scenarios are used in this study (see Table 1 for details).

**Table 1:** Summary of All 82,271 Nonfrozen Ocean Atmospheric Infrared Sounder Observations From 31 October 2018 21:00 UTC to 01 November 2018 21:00 UTC

	Day	Night	Total
|lat| ≤ 30	19,537	17,343	36,880
|lat| in (30,60]	19,263	17,095	36,358
|lat| > 60	2,677	6,356	9,033
All	41,477	40,794	82,271

*Note*. We denote |lat| ≤ 30, |lat| in (30,60], and |lat| > 60 as tropical, midlatitude, and polar zone, respectively.

### 2.2 ECMWF

The model profiles are generated by the ECMWF operational global weather forecasting system (ECMWF, 2009). The best available estimate of the atmospheric state is taken from the short‐range forecast. The ECMWF atmospheric states used by Aumann et al. (2018) were estimated using 3 hr and 0.25° horizontal resolution (∼25 km at the equator) time/space interpolation, which resulted in matchup errors for the comparison with AIRS observations. For this study, we employ the 15 min and 9 km time/space interpolation data used by the ECMWF integrated forecast system. We obtain atmospheric‐state profiles at 137 vertical levels, including pressure, temperature, O_3_, H_2_O, liquid/ice cloud content, and cloud cover.

### 2.3 Community Radiative Transfer Model

CRTM is a fast RT model developed by the Joint Center for Satellite Data Assimilation in the United States (Han et al., 2006). CRTM simulates satellite infrared and microwave radiances with respect to atmospheric‐state variables (e.g., temperature, pressure, humidity, water and ice cloud content, and trace gas concentrations). It consists of the following key modules: gaseous transmission, surface emission and reflection, cloud and aerosol absorption and scattering, and RT solver. The default RT algorithm used for scattering calculations is the Advanced Doubling‐Adding (ADA) algorithm. CRTM also contains a *k*‐matrix module for Jacobian calculations, a tangent‐linear module, and an adjoint module, which are important for radiance assimilation and for the inversion part of retrievals (Garand et al., 2001; Geer et al., 2019; Li et al., 2022; Weng & Liu, 2003).

In this study, we use CRTM version 2.4.0. Its cloud module has six cloud types: water, ice, rain, snow, graupel, and hail, which are defined by the cloud particle densities. We only use water and ice clouds in our calculations. The cloud optical properties (i.e., mass extinction coefficient, single scattering albedo, and asymmetry parameter) data used in this study are taken from the default cloud property lookup table (CloudCoeff.bin, version 3.0.4). The optical properties of water clouds with density less than 0.9 g/cm^3^ are generated based on Mie theory by using a modified gamma distribution (Han et al., 2006). The ice cloud optical properties are based on the MODIS collection 5 (MC5) ice habit model (Baum et al., 2005; Ding et al., 2011). This model chooses a habit mixture scheme (including droxtals, 3D bullet rosettes, hexagonal plates, hollow/solid columns, and aggregates) based on the particle diameter. For individual habits, the optical properties are computed at 65 wavelengths between 0.2 and 100 μm and for 45 size bins.

CRTM also requires the cloud particle effective radius vertical profile. To obtain the effective radius, we use the formulation in Bower et al. (1994) for water clouds and the temperature dependence obtained by Ou et al. (1995, 2013) for ice clouds. Four cloud overlap schemes (maximum overlap, random overlap, maximum‐random overlap, and average overlap) are available in CRTM for all‐sky radiation calculations (van Delst et al., 2016). They use a two‐column radiance approach, which can be expressed by the following formula:

(1) $$R=\left(1-tcc\right)R^{clr}+tccR^{cld}$$

where $R^{clr}$ is the clear sky top of the atmosphere (TOA) radiance, $R^{cld}$ is the cloudy sky TOA radiance (where both water and ice cloud are included), and tcc is the total cloud cover. Note that the values of $R^{cld}$ and tcc are both dependent on the selected cloud overlap scheme. For level *i* in a *n*‐level atmosphere, we denote the cloud cover as $c_{i}$ and the total cloud content for all cloud types as $m_{i}$. Then, tcc can be calculated as follows:

(2a) $$tcc_{max}=max\left(c_{1},c_{2},\text{⋯},c_{n}\right)$$

(2b) $$tcc_{rand}=1-\underset{k=1,n}{\prod }\left(1-c_{k}\right)$$

(2c) $$tcc_{max-rand}=1-\underset{k=1,n}{\prod }\frac{1-\max\left(c_{k-1},c_{k}\right)}{1-c_{k-1}}$$

(2d) $$tcc_{avg}=\frac{\sum_{k}m_{k}c_{k}}{\sum_{k}m_{k}}$$

The maximum‐random overlap scheme in CRTM only uses two columns, possibly reducing its accuracy.

The calculated radiance spectra for the two columns are then combined using Equation 1 to obtain the simulated AIRS radiances using temperature, cloud, water, and ozone profile inputs from ECMWF. The CO_2_ concentration is based on the U.S. 1976 Standard Atmosphere profile but is scaled to 405 ppmv (representative of global mean concentrations for the year 2018). Note that we ignore the seasonal cycle of CO_2_ concentration although its amplitude can be as high as 12 ppmv at some latitudes.

### 2.4 The First Wasserstein Distance

**Figure 1:**
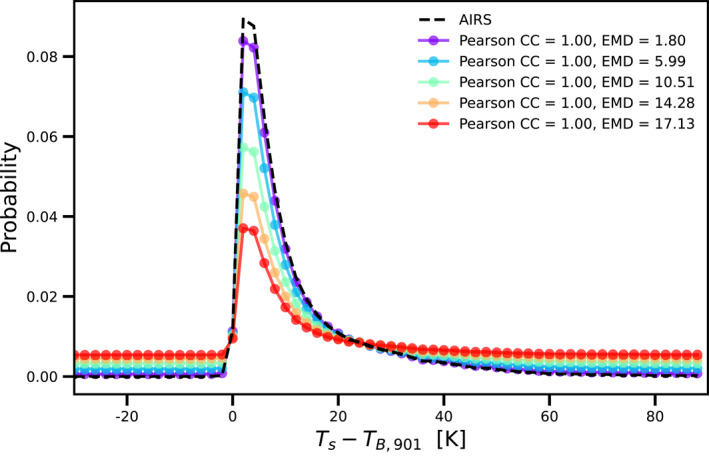
Example of probability distributions that have a Pearson correlation coefficient of 1.0 with respect to the truth and yet are clearly very different. The black‐dashed line is the probability distribution of the difference between ST and brightness temperature in the 901 cm^−1^ channel for the nonfrozen ocean scenarios described in Table 1. The solid colored lines are synthetic scenarios based on linear rescaling of the AIRS probability distribution. The Pearson correlation coefficients and the first Wasserstein distance between the AIRS probability distribution and the solid colored lines are indicated in the legend.

In order to evaluate the performance of the model simulations, we quantitatively measure how far the modeled probability distributions deviate from AIRS observations. More specifically, we analyze probability distributions of the difference between the surface temperature and the channel brightness temperature. Aumann et al. (2018) used the Pearson correlation to compare the model and measurement probability distributions. However, while the Pearson correlation coefficient is a measure of the linear correlation between two variables, it is not suitable for comparing probability distributions. A high degree of correlation does not necessarily imply a high causal relationship. This is illustrated in Figure 1 for a sample AIRS observation. The black‐dashed line is the probability distribution of the difference between the surface temperature and the brightness temperature in the 901 cm^−1^ channel for the nonfrozen ocean scenarios described in Table 1. We do a linear rescaling of the AIRS probability distribution to obtain five synthetic probability distributions (solid colored lines). All the probability distributions shown in Figure 1 have a Pearson correlation coefficient of 1.0 despite clear differences between the distributions and the actual AIRS observations. This degeneracy can be resolved by employing the Wasserstein distance, which is a measurement of the distance between two probability distributions.

The *n*th Wasserstein distance between two distributions p and q is defined as

(3) $$W_{n}\left(p,q\right)={\left(\underset{\gamma \in \Pi (p,q)}{\inf}E_{\left(x,y\right)∼\gamma }[{\left\|x-y\right\|}^{n}]\right)}^{1/n}\text{,}$$

where Π(p,q) is the collection of all possible joint distributions between p and q. *x* and *y* are a pair of random vectors marginally distributed as p and q. *inf* (infimum) represents the greatest lower bound. For each possible joint distribution γ∈Π(p,q), we calculate the expected value of the distance for all pairs of samples from p to q. The lower bound is the *n*th Wasserstein distance.

The first Wasserstein distance is also known as the Earth Mover's Distance (EMD), which has important applications in the field of computer science (Arjovsky et al., 2017; Rubner et al., 2000). Intuitively, if one thinks of two probability distributions as two different ways of piling up a certain amount of dirt, then EMD is the minimum cost of turning one pile into the other, where cost is assumed to be the product of the amount of dirt moved and the distance by which it is moved.

Assume that the lengths of two discrete distributions p and q are *M* and *N*, respectively. Further, $\omega_{i}$ and $\omega_{j}\;$ are values at locations i and j within those respective distributions. The first Wasserstein distance can then be calculated using the following formula:

$$W\left(p,q\right)=\underset{\forall f}{\text{inf}}\frac{\sum_{i=1}^{M}\sum_{j=1}^{N}d_{ij}f_{ij}}{\sum_{i=1}^{M}\sum_{j=1}^{N}f_{ij}},\;$$

$$s.t.\;f_{ij}\geq 0,\;for\;1\leq i\leq M,\;1\leq j\leq N$$

$$\overset{N}{\underset{j=1}{\sum }}f_{ij}\leq \omega_{i},\;for\;1\leq i\leq M$$

$$\overset{M}{\underset{i=1}{\sum }}f_{ij}\leq \omega_{j},\;for\;1\leq j\leq N$$

(4) $$\overset{N}{\underset{j=1}{\sum }}\overset{M}{\underset{i=1}{\sum }}f_{ij}=min\left(\overset{M}{\underset{i=1}{\sum }}\omega_{i},\overset{N}{\underset{j=1}{\sum }}\omega_{j}\right)$$

where $d_{ij}$ is the distance between $p_{i}$ and $q_{j}$, and f is any possible way to move p to q. $f_{ij}$ is the amount that should be moved from $p_{i}$ to $q_{j}$.

In our study, we use the python package “SciPy” to calculate the first Wasserstein distance. Figure 1 shows the first Wasserstein distance between AIRS observations and synthetic probability distributions; the utility of the first Wasserstein distance as a metric is clearly evident.

There are other statistical metrics for comparing probability distributions or histograms, such as Kullback‐Leibler (KL) divergence and neighborhood statistics (Geer & Baordo, 2014; Roberts & Lean, 2008). While there are mathematical links between the first Wasserstein distance and the KL divergence (Amari et al., 2018), the advantage of the former is that it is symmetric (W(p,q)=W(q,p)). On the other hand, the neighborhood statistics method requires conversion of the raw data to binary fields based on some threshold filter, while the first Wasserstein distance is a nonparametric metric that does not lose information through the application of any filter. Furthermore, the first Wasserstein distance is a natural way to compare two probability distributions where one is derived from the other by making small perturbations.

## 3 Results

Table 1 summarizes the scenarios used in this study. We denote |lat| ≤ 30, |lat| in (30,60), and |lat| > 60 as tropical, midlatitude, and polar zone, respectively. Daytime and nighttime cases are roughly balanced in the data set. Both the tropical and midlatitude zone scenarios account for ∼44% of the 82,271 cases. However, as we select only nonfrozen scenarios and because the majority of the high‐latitude region is covered by ice, only ∼11% of the chosen scenarios are in the polar zone.

**Figure 2:**
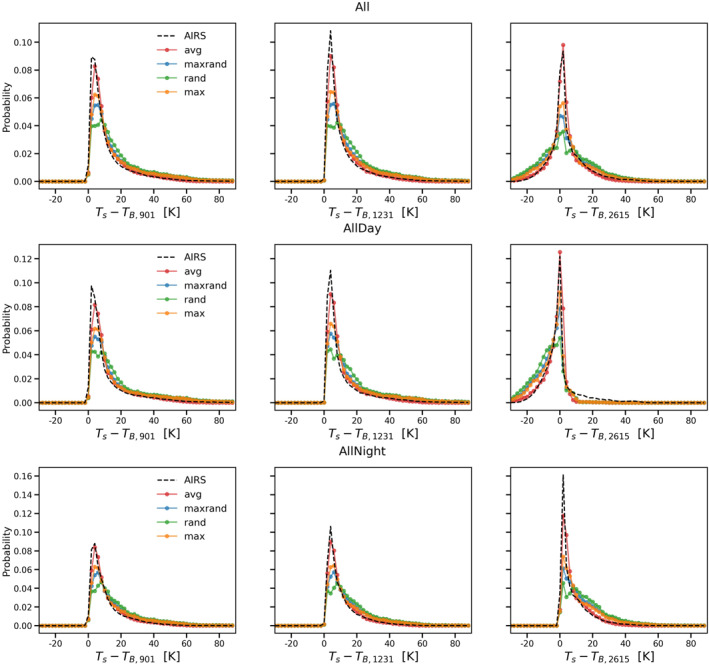
Probability distributions of the difference between ST and brightness temperature in three atmospheric window channels. (901 cm^−1^, 1231 cm^−1^, and 2615 cm^−1^) for the nonfrozen ocean day and night scenarios. The AIRS observations are shown in dashed black, and the corresponding CRTM simulation results using the average overlap, maximum‐random overlap, random overlap, and maximum overlap schemes are shown in red, blue, green, and orange, respectively.

In this study, we first choose three atmospheric window channels (901 cm^−1^, 1231 cm^−1^, and 2615 cm^−1^). The first two are in the thermal infrared spectral range, while the last one is in the shortwave infrared (where effects due to reflection and scattering of solar radiation need to be considered). Figure 2 shows probability distribution plots of the difference between surface temperature (ST) and channel brightness temperature (T_B_) for AIRS observations (dashed black) and CRTM with average overlap (red), maximum‐random overlap (blue), random overlap (green), and maximum overlap (orange) schemes in these three channels for nonfrozen ocean day and night cases. On each curve, there are 60 points from −30 to 90 K with a 2 K temperature interval. The peak of the probability distribution in the infrared channels (901 cm^−1^ and 1,231 cm^−1^) is near 5 K, which indicates relatively little cloudiness or a clear‐sky scenario. We only observe daytime cases in the 2,615 cm^−1^ channel with negative (ST–T_B_) values, showing surface reflection effects or strong forward scattering from the clouds. CRTM with average overlap not only successfully simulates the peak of the probability distribution for all scenarios but also reproduces the overall shape of the observed distribution. In contrast, the other overlap options produce worse results, especially with respect to the peak, with maximum overlap performing the best and random overlap the worst. The random overlap scheme places all vertical subgrid clouds in a random fashion, usually resulting in a higher tcc compared to the other cloud overlap schemes. Previous studies have also found that the random overlap scheme performs poorly for clouds in the lower troposphere (see, e.g., Hogan and Illingworth (2000)). Overall, random overlap, maximum‐random overlap, and maximum overlap overestimate the total cloud fraction, resulting in a smaller brightness temperature compared with observations.

Table 2 summarizes the first Wasserstein distances as well as the Pearson correlation coefficients of the simulated radiances in Figure 2 against the observations. The models with best performance for each scenario in Table 2 are marked in red. Note that a smaller first Wasserstein distance or a larger Pearson correlation coefficient indicates better agreement with observations. In almost every scenario, CRTM with the average overlap provides the best results based on both the first Wasserstein distance and the Pearson correlation coefficient. The only exception is daytime at 2,615 cm^−1^. For this scenario, the Pearson correlation coefficient suggests that the maximum overlap scheme is the best, while the first Wasserstein distance indicates that the average overlap scheme matches the observations better than the other options. From the daytime all zone 2,615 cm^−1^ subplot in Figure 2, it is evident that the average overlap results (red) provide the best match with the AIRS observations. The maximum overlap simulations produce many more cases with (ST–T_B_) between −20 K and −5K, and fewer cases for the peak near 0 K. This suggests that the maximum overlap scheme underestimates low cloud effects. This special case indicates that the first Wasserstein distance is a better metric than the Pearson correlation coefficient for comparing two probability distributions.

**Table 2:** Comparison of Community Radiative Transfer Model Performance With Different Overlap Schemes for All (Day and Night), Day Only, and Night Only Scenarios in Three Window Channels Using Two Metrics: Pearson Correlation Coefficient and First Wasserstein Distance

	901 (Pearson)	1231 (Pearson)	2615 (Pearson)	901 (EMD)	1231 (EMD)	2615 (EMD)
**All (Day and night)**						
Avg	0.97	0.98	0.99	1.09700	1.03078	1.45737
Maxrand	0.94	0.92	0.93	5.82570	5.60864	3.51647
Rand	0.87	0.83	0.84	7.30072	7.25629	5.28872
Max	0.95	0.95	0.96	4.82662	4.52302	2.49286
**Day only**						
Avg	0.96	0.96	0.95	1.38527	1.43431	2.94494
Maxrand	0.92	0.92	0.94	6.12437	5.89860	5.64161
Rand	0.86	0.84	0.83	7.55617	7.52913	7.05433
Max	0.94	0.87	0.98	5.12481	4.79064	4.97815
**Night only**						
Avg	0.98	0.99	0.94	0.94442	0.62352	0.99065
Maxrand	0.94	0.93	0.84	5.52464	5.31558	5.99179
Rand	0.87	0.83	0.72	7.04408	6.98068	8.10498
Max	0.96	0.96	0.88	4.52577	4.25268	4.63390

*Note*. The best performing models for each channel and metric are marked in red.

**Figure 3:**
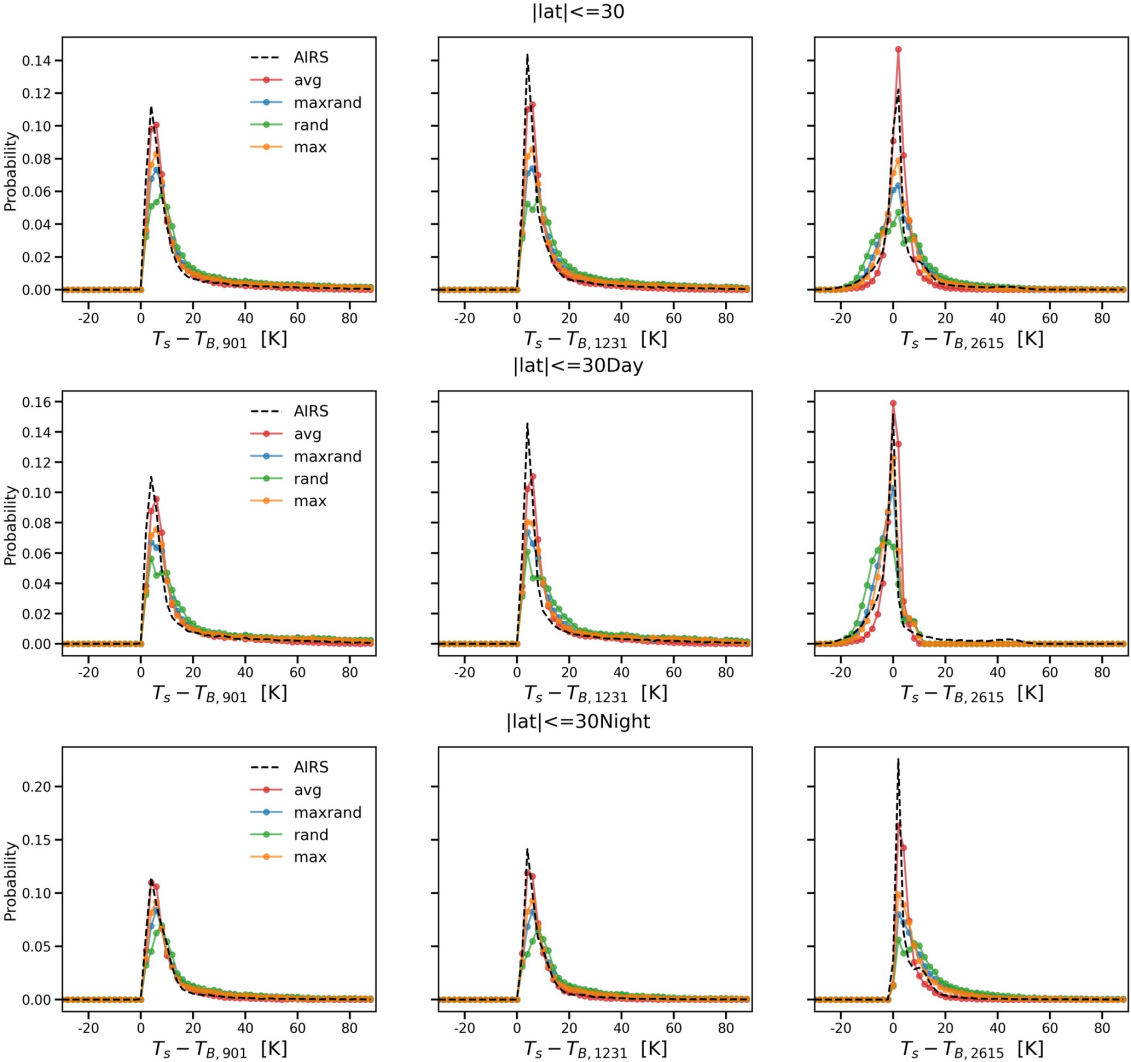
Same as Figure 2 but for tropical zone only.

**Figure 4:**
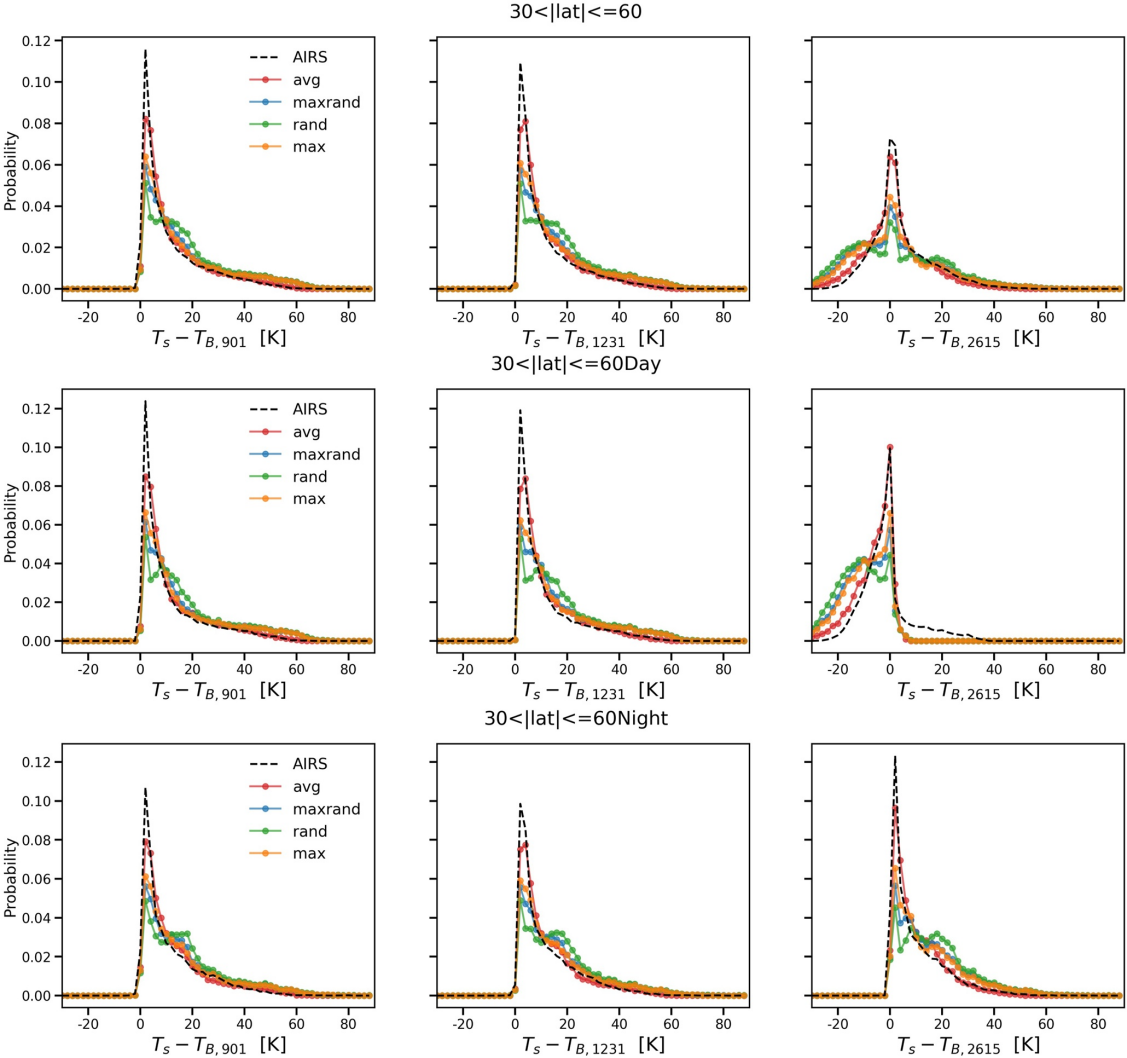
Same as Figure 2 but for midlatitude zone only.

**Figure 5:**
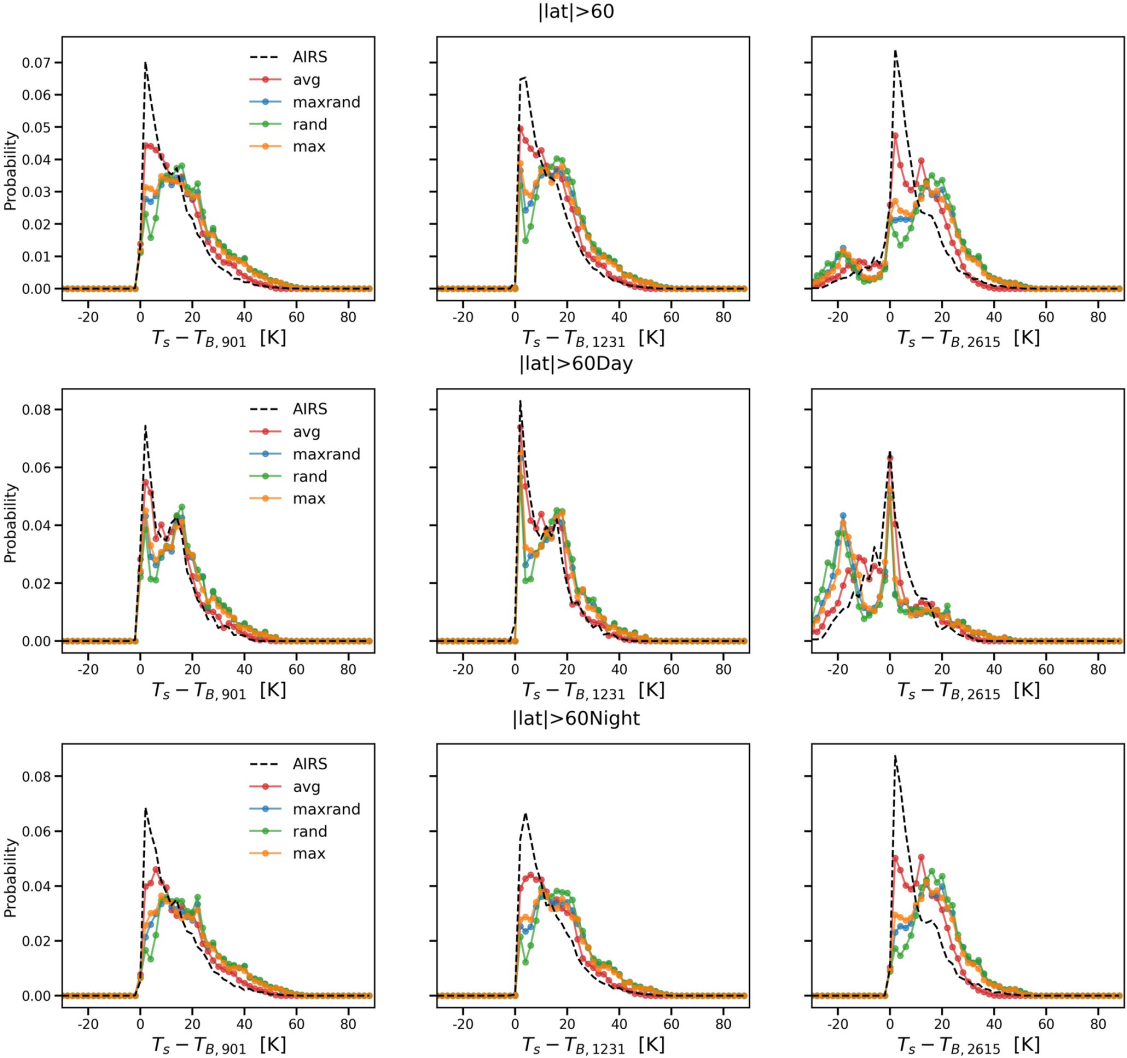
Same as Figure 3 but for polar zone only.

Figures 3, 4, 5 are similar to Figure 2 but for tropical (|lat|≤30), midlatitude (30<|lat|≤60), and polar (|lat|>60) zones, respectively. Overall, the average overlap scheme still gives the best results among the four options, reproducing both the peak and the shape of the probability distributions with high fidelity. The model performance is relatively worse in the polar zone compared to the other two zones (Figure 5). Although all four cloud overlap schemes are able to simulate the two peaks for the daytime scenario in the polar zone, the secondary peak for this case has a negative bias (of nearly 5 K compared to AIRS observations). The models also fail to match the nighttime scenario peak for the polar zone in all three window channels. The simulations are colder than the actual AIRS observations, indicating that the cloud optical depth is overestimated and/or the clouds are placed too high in the simulations.

**Figure 6:**
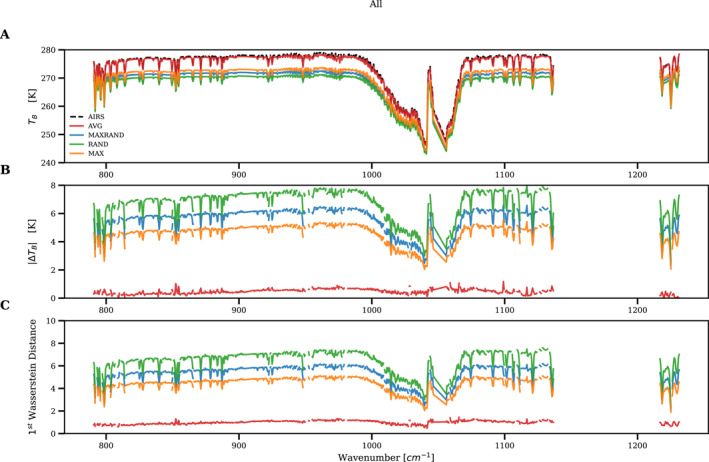
(a) Mean brightness temperature for, (b) mean absolute brightness temperature difference between, and (c) first Wasserstein distance between CRTM radiance simulations and AIRS observations for 846 AIRS channels between 790 cm^−1^ and 1231 cm^−1^. Channels with noise >1 K have been removed before performing these calculations. The color scheme is the same as in Figure 2.

In addition to the three window channels, we also evaluate the simulated radiances using a wide range of channels that cover CO_2_, H_2_O, and O_3_ absorption regions. In this analysis, the mean T_B_, mean absolute T_B_, and the first Wasserstein distance with respect to AIRS observations are compared. Results are shown in Figure 6. Again, average overlap simulations show very good agreement with observations (T_B_ discrepancies are less than 0.7 K) for all 846 channels, including the O_3_ absorption band around 1,040 cm^−1^.

**Figure 7:**
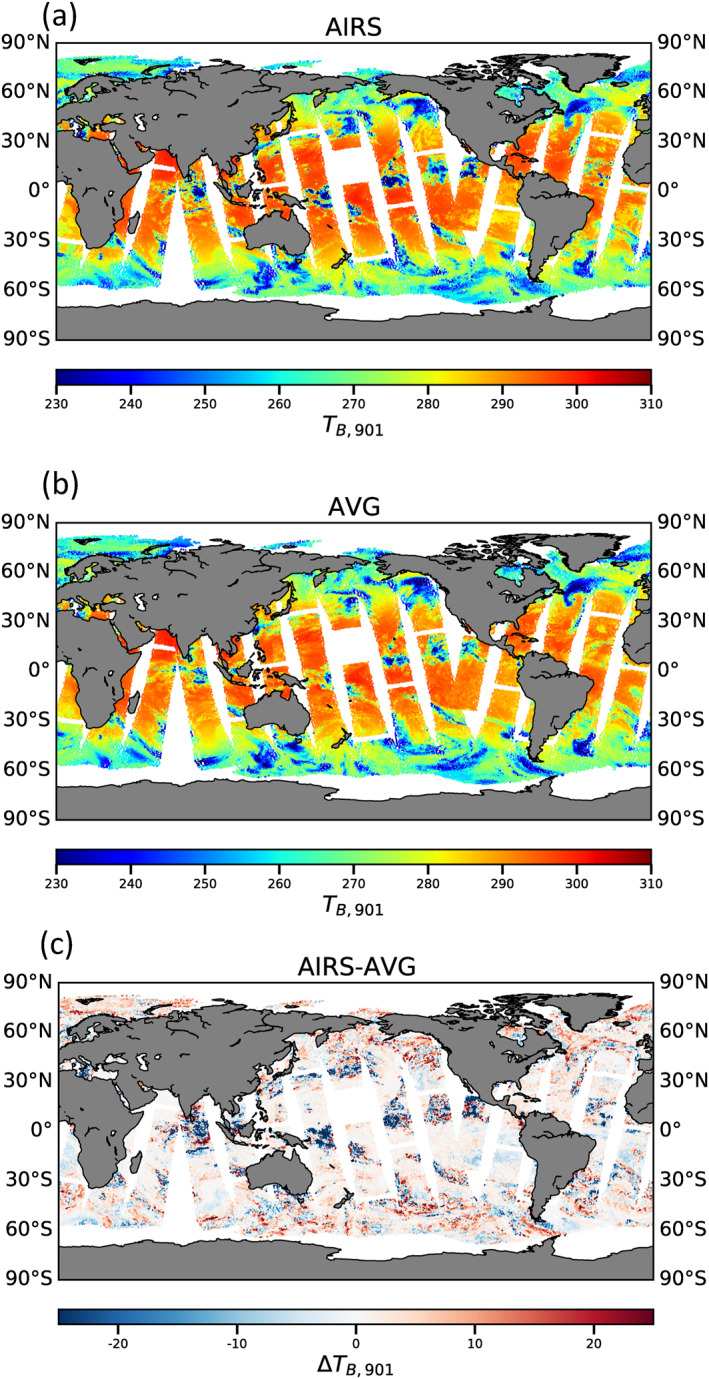
Visualization of brightness temperature at 901 cm^−1^ for all scenarios for (a) AIRS observations, (b) CRTM with average overlap scheme, and (c) difference between AIRS observations and CRTM with average overlap scheme.

Figure 7 illustrates the pairwise comparison of observed AIRS 901 cm^−1^ T_B_ with CRTM average overlap simulations on a global map. In general, the simulated radiances agree well with AIRS observations on a global scale; the absolute difference is less than 2 K in most cases. However, a clear regional pattern is evident, especially for the Intertropical Convergence Zone (ITCZ) where the absolute difference can be greater than 30 K.

## 4 Conclusions and Discussion

In this study, we employ CRTM with four different cloud overlap schemes to calculate the TOA radiation field in three atmospheric window channels and several hundred absorbing channels (covering the thermal and shortwave infrared spectral regions) for a large number of cloudy atmospheric scenarios and compare the results against AIRS observations using the first Wasserstein distance as a statistical metric. We use atmospheric‐state profiles from ECMWF at 137 vertical levels as the inputs for CRTM. Our results show that the average overlap scheme successfully predicts the overall probability distribution of clouds over a wide range of spectral channels between 790 cm^−1^ and 1,231 cm^−1^ for over 80,000 scenarios.

CRTM simulations have zone‐dependent biases, especially for the polar region. In particular, it has difficulty reproducing the secondary peak of the probability distribution function for the 2,615 cm^−1^ channel. Moreover, a pairwise comparison between CRTM average overlap simulations and AIRS radiances shows a regional pattern. There are three possible error sources in our simulations: (a) random and systematic error from ECMWF cloud profiles (e.g., water/ice cloud content and cloud cover), (b) error due to oversimplified assumptions about cloud overlap, and (c) error from cloud optical property coefficients. Our approach of comparing the overall probability distribution functions between model and observation can mostly cancel out the random errors in the ECMWF profiles but will not eliminate systematic biases.

Further investigations are required to evaluate the best cloud overlap assumption for different scenarios and spatial scales. The first Wasserstein distance is a superior statistical metric (compared to traditionally used metrics such as the Pearson correlation coefficient) to evaluate the fidelity of RT models with respect to observations and should be used in future RT model intercomparisons. Another approach could be to use a cloud resolving model (CRM) to create a large number of scenarios with different vertical cloud distributions with a statistical analysis, which then performed to compare against simulations using the multiple independent column approximation (ICA) simulations that employ the CRM cloud fields as the “truth.” ICA provides the best physical reference as long as sufficient independent columns are used. It is also critical to minimize the spatiotemporal mismatch between ECMWF and AIRS locations. Clouds vary a lot in space and time and it is very easy for biases to creep in simply because of errors in characterizing a scene as clear when it is cloudy and vice versa. Comparisons with ICA simulations eliminate the issue of model errors and allow usage of more conventional statistical approaches to evaluate the performance of the two‐column overlap schemes for different scenarios.

The importance of such studies is clearly stated in the recently released Earth Science Decadal Survey (National Academies of Sciences, Engineering, and Medicine, 2018), which recommends a set of observation capabilities that will enable substantial progress in (a) providing critical information on the makeup and distribution of clouds and (b) addressing key questions about how changing cloud cover and precipitation will affect climate, weather, and Earth's energy balance in the future.

## Data Availability

The AIRS observations and ECMWF vertical profiles used in this study can be downloaded from https://airsteam.jpl.nasa.gov/ftp/hha/ECMWF20181101/. Figures were made with Matplotlib version 3.0.2 (Caswell et al., 2018; Hunter, 2007), available under the Matplotlib license at https://matplotlib.org/. The first Wasserstein distances were calculated using Scipy v1.0 (Virtanen et al., 2020), available at https://scipy.org/. The RT code used in this manuscript (CRTM version 2.4.0) is licensed under CC0 and published on Github: https://github.com/JCSDA/crtm.
